# Recent Progress in Using Mesoporous Carbon Materials as Catalyst Support for Proton Exchange Membrane Fuel Cells

**DOI:** 10.3390/nano13212818

**Published:** 2023-10-24

**Authors:** Guanxiong Wang, Wei Zhao, Majid Mansoor, Yinan Liu, Xiuyue Wang, Kunye Zhang, Cailin Xiao, Quansheng Liu, Lingling Mao, Min Wang, Haifeng Lv

**Affiliations:** 1 Shenzhen Academy of Aerospace Technology, Shenzhen 518057, China; wanggx131@126.com (G.W.); xiaocl@mail.sustech.edu.cn (C.X.); 15303141535@163.com (Q.L.); 2 College of New Energy, China University of Petroleum (East China), Qingdao 266580, China; s22030109@s.upc.edu.cn (W.Z.); 2115050124@s.upc.edu.cn (Y.L.); s23150051@s.upc.edu.cn (X.W.); 2203050220@s.upc.edu.cn (K.Z.); 3 College of Energy Soochow, Institute for Energy and Materials Innovations, Soochow University, Suzhou 215006, China; 20227240002@stu.suda.edu.cn; 4 Department of Chemistry, Southern University of Science and Technology, Shenzhen 518055, China; maoll@sustech.edu.cn

**Keywords:** mesoporous carbon, intermetallic, oxygen reduction reaction, mass transport resistance, durability

## Abstract

Developing durable oxygen reduction reaction (ORR) electrocatalysts is essential to step up the large-scale applications of proton exchange membrane fuel cells (PEMFCs). Traditional ORR electrocatalysts provide satisfactory activity, yet their poor durability limits the long-term applications of PEMFCs. Porous carbon used as catalyst support in Pt/C is vulnerable to oxidation under high potential conditions, leading to Pt nanoparticle dissolution and carbon corrosion. Thus, integrating Pt nanoparticles into highly graphitic mesoporous carbons could provide long-term stability. This Perspective seeks to reframe the existing approaches to employing Pt alloys and mesoporous carbon-integrated ORR electrocatalysts to improve the activity and stability of PEMFCs. The unusual porous structure of mesoporous carbons promotes oxygen transport, and graphitization provides balanced stability. Furthermore, the synergistic effect between Pt alloys and heteroatom doping in mesoporous carbons not only provides a great anchoring surface for catalyst nanoparticles but also improves the intrinsic activity. Furthermore, the addition of Pt alloys into mesoporous carbon optimizes the available surface area and creates an effective electron transfer channel, reducing the mass transport resistance. The long-term goals for fuel-cell-powered cars, especially those designed for heavy-duty use, are well aligned with the results shown when this hybrid material is used in PEMFCs to improve performance and durability.

## 1. Introduction

**Figure 1:**
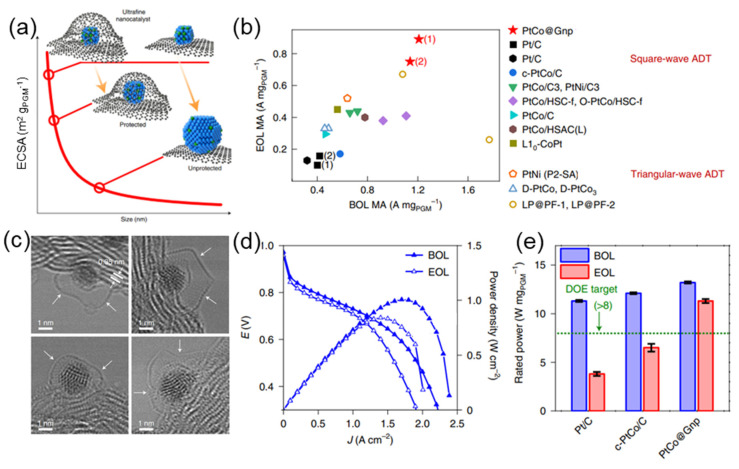
(**a**) Schematic illustration of ultrafine nanocatalysts encaged in graphene pockets and their impact on ECSA retention after an ADT. (**b**) Comparison of mass activity between PtCo catalysts with the state of the art in the literature. (**c**) The enclosure of ultrafine nanoparticle by graphene nanopockets (indicated by white arrows). (**d**) Comparison of PtCo@Gnp at BOL and EOL. (**e**) Rated power of MEAs normalized by the total PGM loading [3].

Recently, large-scale applications of proton exchange membrane fuel cells (PEMFCs) have sparked a meteoric rise in fuel cell technology. Among the forerunners in this field is Toyota’s Mirai fuel cell vehicle, which features a stunning improvement in the volumetric power density of its stack, from 3.5 kW/L in its first iteration to 5.4 kW/L in its second [1]. Because of this 41.9% improvement in performance measures, the Mirai is now among the top products in terms of volumetric power density in the world [2]. However, the catalyst system based on mesoporous carbon (MPC) embedded with PtCo alloy nanoparticles is what makes this advancement genuinely ground-breaking (Figure 1). In contrast to the first-generation Mirai’s use of Pt distributed on conventional solid carbon supports, the second-generation system uses a novel combination of PtCo alloy nanoparticles with MPC scaffolds [3].

The novel feature resides in the MPC complex design, allowing for the uniform distribution of PtCo nanoparticles and neatly accommodating them within its mesoporous channels. This configuration’s unique benefits include reducing the ionomer’s interaction with the Pt surface and avoiding the poisoning impact of the sulfonic acid group. Additionally, it improves the mass-specific activity of the Pt catalysts, ushering in a new era of fuel cell efficiency while simultaneously decreasing Pt consumption [4]. The PEMFC ecosystem has been rethought from the ground up, providing a model for the future of environmentally friendly, fuel-efficient transportation [5]. The new Mirai is more than just an improvement; it is a technological leap that, thanks to advances in materials science, ushers in a new era for hydrogen fuel cell automobiles [6]. Though the overarching goal on both sides of the Pacific is to improve fuel cell technology, a new story is emerging about the significance of MPC supports and Pt nanowires in redefining ORR kinetics and catalyst longevity, particularly at low Pt loadings [7,8].

This review takes a novel approach to dissecting how the unique structure of MPC serves as protection against Pt poisoning while simultaneously facilitating local oxygen transfer. We highlight the integration of MPC, a novel departure from prior work, to meet the demanding criteria of high-power fuel cell applications. Standard catalyst systems are insufficient due to their severe 25,000 h durability criterion and their limited Pt loading of 0.3 mg/cm^2^ [9]. Therefore, nanoscale structural and chemical reengineering must replace simple performance enhancement as the primary design goal for catalysts. Given this context, this paper provides an overview of cutting-edge methods for synthesizing MPCs that meet these demanding requirements and considerably contribute to lowering the kinetic overpotential. In what follows, we will discuss the most cutting-edge techniques for depositing PtCo intermetallics selectively onto MPC substrates. This major breakthrough holds great promise as a key to developing the next generation of ORR catalysts that will set the standard for performance and durability. In conclusion, this work can be used as a guide for developing MPC supports and PtCo-based catalysts that not only meet but surpass the lofty targets established for the future of fuel cell applications, especially in heavy-duty trucks. The groundwork laid here paves the way for a new, more efficient, sustainable, and robust era in fuel cell technology.

## 2. MPC in PEMFC Catalysts

In fuel cell technology, navigating complex carbon support systems is analogous to trying to thread a needle. MPC has been identified as a significant advancement in optimizing catalytic activity and long-term stability [10]. This substance exhibits a composition extending beyond pure carbon, encompassing intricate structures characterized by disordered and ordered networks. Additionally, it possesses a diverse variety of pore sizes, ranging from 2 to 50 nm [11]. The distinctive structural peculiarity of MPC confers it with a notable advantage in addressing the inherent restrictions that have hitherto hindered fuel cell catalysts [12]. However, why limit ourselves to solely enhancing catalyst activity? The primary difficulty involves protecting the Pt catalyst from the deleterious effects of sulfonate groups found in perfluoro sulfonic acid ionomers (PFSAs), which have been seen to reduce the activity of Pt by a significant factor of 2–4 [13]. 

Catalysts used in PEMFCs are profoundly affected by the structure and morphology of the carbon supports used in their design and performance [14]. One must be aware of the potential “poisoning” effects of sulfonic acid groups, especially from perfluoro sulfonic acid ionomers, on Pt activity, even though there are many methods for tuning the intrinsic activity of catalysts [15]. Standard porous carbon supports enable the deposition of Pt nanoparticles within their pore structures, reducing their interaction with sulfonic acid groups. However, these supports present difficulties in the mass transfer of reaction gas (O_2_) and conduction of protons (H^+^), which limit their usefulness [16].

The aforementioned constraints can be a significant impediment, particularly in areas with high current density, which is important in heavy-duty vehicles [17]. The unique contributions of MPC are significant in providing an optimal compromise [18]. The distinctive architectural and electronic characteristics of MPC render it an exceedingly auspicious substrate for PtCo intermetallics [19]. MPC is a supplementary material that effectively integrates the catalytic function and electrochemical kinetics within PEMFCs. The utilization of this technique leads to an increase in the stability and visibility of active sites responsible for the ORR [20]. Additionally, it greatly enhances the transportation of oxygen inside the catalyst layer. As a result, this approach effectively tackles the drawbacks associated with conventional carbon supports. The anti-corrosion and anti-oxidation capabilities of MPC supports have been significantly improved by the progress made in surface functionalization, graphitization, and heteroatom doping [21]. Furthermore, novel techniques, such as high-temperature annealing, have been devised to enhance the durability and effectiveness of these MPC supports, especially when subjected to rapid stress testing [22].

**Figure 2:**
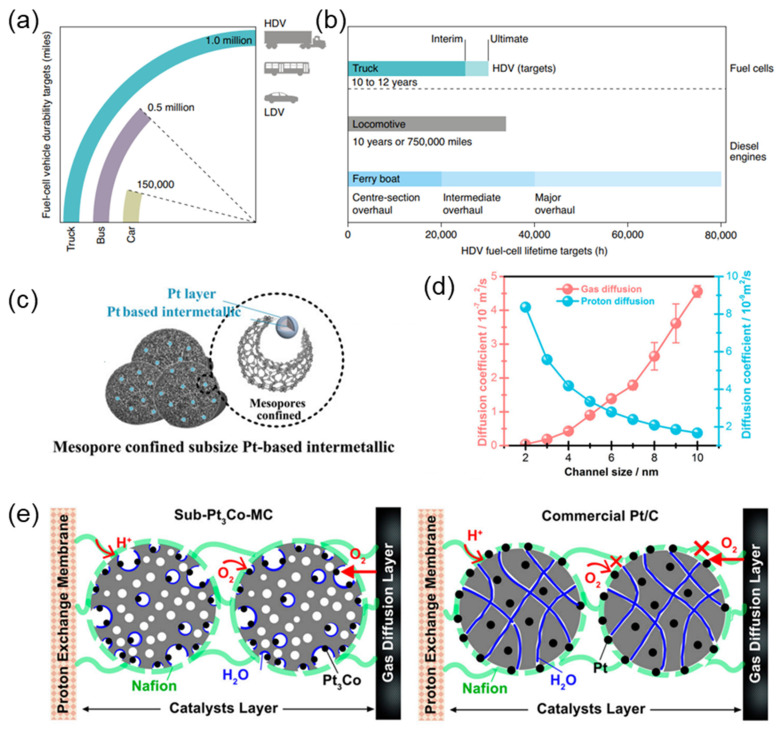
(**a**) Fuel cell durability targets for LDVs and HDVs expressed in terms of miles. (**b**) Comparison of HDV fuel cell lifetime targets with the useful service lifetime of current diesel engines for rail [26]. (**c**) Structure of Pt-M intermetallic–Pt skin with mesopore confined. (**d**) The proton and gas diffusion capacity. (**e**) The anti-poisoning effect with three-phase interface in sub-Pt_3_Co-MC and the poison effect by Nafion ionomer in commercial Pt/C [16].

By merging the inherent benefits of MPC with PtCo intermetallics, it becomes possible to address the difficulties related to Pt poisoning, mass transfer resistance, and proton conduction. The collaboration between MPC and PtCo intermetallics demonstrates a possible avenue for achieving the desired long-term performance and durability objectives of PEMFCs, specifically in the challenging and dynamic domain of heavy-duty vehicles (Figure 2a,b) [23,24,25,26,27]. Hence, the significance of employing MPC support in augmenting the efficiency of PEMFCs becomes apparent when considering the broader framework encompassing catalyst durability, reactivity, and operational complexities. The scaffold is not solely a passive structure but rather an active entity that plays a vital role in influencing both catalyst kinetics and mass transport phenomena (Figure 2c–e) [16].

To make breakthroughs in fuel cell technology, researchers need a deep familiarity with the intricate relationships between catalysts, supports, and operating parameters. Protecting Pt nanoparticles from sulfonic acid groups with conventional MPC catalysts can maintain their catalytic activity. This benefit, however, comes at the expense of stifling O_2_ and H^+^ mass transfer efficiency, especially in high-current-density regions above 2.0 A/cm^2^ [28]. The appropriate carbon support should be more than merely a safe habitat for Pt if high performance is to be achieved, especially at a modest platinum loading (10 g/vehicle or 0.1 mg_Pt_/cm^2^ MEA). 

Combining nano-architecture channels for the unrestricted flux of H^+^ and O_2_ with micro-scale isolation of Pt nanoparticles from ionomers should be a feat of engineering. Using MPC supports that are functionally graded, with different pore shapes at different depths, is a novel method. Such hierarchical arrangements would provide the optimal compromise between preserving mass transfer efficiency and isolating Pt nanoparticles. These graded supports can potentially optimize pore depth and tortuosity for each application, whereas in conventional MPC, these factors may limit performance. Imagine an MPC architecture with multiple layers, each serving a different purpose, such as one layer for optimal Pt nanoparticle implantation, another for quick proton transfer, and still another tuned for maximum O_2_ diffusion. By strategically engineering these functionally graded layers, we can overcome obstacles like catalyst poisoning and mass transfer limits, satisfying demands for increased activity and longer lifetimes [29,30]. 

Although the concept is rather simple, the severe inhomogeneous nature and the size of the carbon black particles make visualization and development of the carbon difficult. Several methods have been developed for the synthesis of MPC, including (1) activation methods (physical activation and chemical activation), (2) template methods (hard template and soft template) [31], (3) carbonized method (the carbonization of polymer/polymer blends, organic aerogels, etc.), and (4) catalytic activation using metal ions. Currently, the “template method” is mainly used to prepare MPC. The carbon precursor is attached to the template material, such as silica, magnesium oxide (MgO), etc., and after high-temperature heat treatment, the templating agent removed via etching, forming a carbon support with a mesoporous pore structure [32,33,34]. Recently, the synthesis procedure for mesoporous carbons using magnesium oxide as a template has been industrialized [35].

For catalysis, new studies highlight the synergistic potential of mesoporous carbon supports with PtCo nanoparticles. Three prominent advantages are as follows: (1) The utilization of advanced technology enables the efficient and uniform distribution of PtCo nanoparticles within the mesoporous carbon matrix. (2) The placement of PtCo nanoparticles within the mesopores effectively isolates them from sulfonic acid groups, resulting in improved kinetics for catalyst reactions involving H^+^ and O_2_. (3) Integrating these state-of-the-art MPC-supported catalysts into electrode assemblies leads to reduced resistance in O_2_ mass transfer and exceptional performance in high-current-density conditions. Consequently, the power density of PEMFCs is significantly enhanced. 

Hydrogen has a high gravimetric energy density, making it ideal for heavy-duty applications such as ships and trains. This is changing the economic dynamics around hydrogen-based technologies, which were previously focused on light-duty vehicles. The cost of ownership has become a significant concern due to trucks needing more durable and cost-efficient fuel cells that fulfill higher criteria than those used in light-duty vehicles. The game-changing potential resides in integrating MPC substrates with advanced Pt alloy production processes, enabling the engineering of catalysts with unparalleled reaction efficiency. The use of this synergistic strategy not only enhances the performance metrics but also presents a persuasive argument for the cost-effective implementation of robust hydrogen fuel cell systems [26].

## 3. Pt Alloy Embedded into Mesoporous Carbon

Recent years have seen an increased focus on developing highly active and long-lasting Pt-based electrocatalysts to drive the widespread industrial use of fuel cells [2]. In addition to controlling particle size and dispersion, the effectiveness of Pt in the ORR can be enhanced by the deliberate incorporation of transition metals such as cobalt and nickel into its alloy structure [36]. However, inside the corrosive environment of PEMFCs, de-alloying can easily occur, leading to catalyst degradation [37].

Two novel approaches to enhancing the stability of alloys have been identified. The initial strategy involves the implementation of Pt shell coating onto the alloy to achieve a core–shell structure [38]. Jong-Sing Yu et al. [39], in their research, presented a recently developed catalyst in the form of a one-dimensional nanowire. This nanowire catalyst consists of an intermetallic PtCo alloy core with an L10-ordered structure. The core is surrounded by a Pt-rich shell that exhibits high-index facets and is under compressive strain. This study was referenced by Tetteh et al. [40]. The combination of sub-surface cobalt-induced strain with a high Miller index alignment and oxygen on the step sites reveals domains with a high activity level for the ORR. As a result, the PtCo alloy exhibits significant endurance. This observation provides additional support for the hypothesis proposed in the technical roadmap, which argues that the development of Pt nanowires has the potential to greatly reduce reaction resistance, hence resulting in remarkable efficiency in the turnover of the ORR.

The alternate stabilizing technique revolves around manipulating the atomic arrangement inside the alloy framework. High-temperature annealing has been identified as an effective method to promote the systematic arrangement of atoms inside intermetallic compound structures. Nevertheless, the increased temperature can potentially induce the sintering process of PtCo nanoparticles [41]. Using mesoporous carbon has emerged as a promising solution to address this particular difficulty. Recently, PtCo alloy integrated onto carbon nanotubes has shown a huge activity boost with remarkable stability [42]. The synergistic integration of PtCo into mesoporous carbon nanotubes demonstrates that the contribution of alloy components and structure in the platinum–carbon-integrated catalyst is responsible for the high-efficiency ORR in fuel cells. Such a hybrid structure results in booting the intrinsic activity of Pt-C architecture [43].

**Figure 3:**
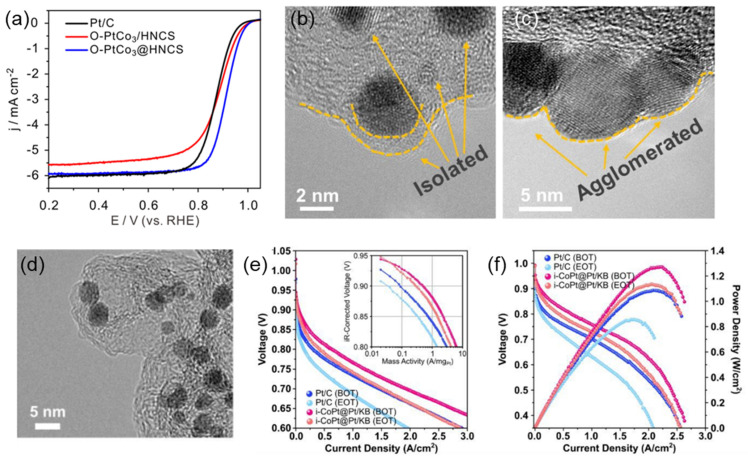
(**a**) LSV curves in O_2_-saturated 0.1 M HClO_4_ solution at room temperature, with a scan rate ~10 mV/s. (**b**,**c**) TEM images at different magnifications of O-PtCo_3_@HNCS and O-PtCo_3_/HNCS [31]. (**d**) TEM image of i-CoPt@Pt/KB, (**e**) H_2_-O_2_ fuel cell polarization curves, and the derived Tafel plots (inset) before and after ADT cycling. (**f**) H_2_-air fuel cell polarization and power density curves of i-CoPt@Pt/KB and Pt/C before and after ADT cycling [45].

MPC is essential because it promotes the synthesis of a structured arrangement of PtCo atoms, preventing nanoparticle aggregation. By employing the impregnation methodology in conjunction with high-temperature post-treatment technology, it is possible to synthesize Pt alloy nanocatalysts that exhibit well-organized atomic structures (Figure 3a–c). This synthesis approach capitalizes on the utilization of MPC supports, which are present both within the pore architecture and on the surface [44]. Nevertheless, achieving increased treatment temperatures up to 1000 °C presents a formidable obstacle. Under heat conditions, the likelihood of PtCo nanoparticle sintering increases, leading to a subsequent decrease in the bulk activity of the catalyst. Ex situ X-ray diffraction research elucidates that Pt and Co ions within the carbon matrix merge to construct Pt-rich alloys at a low temperature of 300 °C, which then evolve into Co clusters at a higher temperature of 500 °C. The morphological evolution persists as the temperature increases to 800 °C, transforming the composition into random alloy nanoparticles of CoPt. Subsequently, at a critical point after 6 h, these nanoparticles undergo a metamorphosis into i-CoPt nanoparticles when the temperature reaches 900 °C. Interestingly, when subjected to a current density of 0.8 A cm^−2^, i-CoPt@Pt/KB exhibits a significant decrease in cell voltage of 29 mV before and after accelerated durability testing. This sharply contrasts with the 73 mV reduction observed in commercial Pt/C counterparts. In addition, the i-CoPt@Pt/KB catalytic ensemble demonstrates significant progress in fuel cell catalytic technology, achieving a power density peak of 1.18 W cm^−2^ at a pragmatic working voltage benchmark of 0.67 V, which has been tailored for a high power yield (Figure 3d–f) [45].

In the first step toward developing a robust PtCo alloy for ORR, researchers used mesoporous carbon as a nano-reactorial region [46]. Synthesized short-ordered mesoporous carbon can simultaneously facilitate the formation of Pt nanoparticles without sintering, even at a high temperature of 900 °C. Additionally, it provides a reduced meso-channel that is suitable for the impregnation of metallic precursors. By carefully adjusting the annealing temperatures and precursor ratios, a collection of PtCo alloys was synthesized, exhibiting similar surface properties but differing structure and composition. An observable transformation in the electronic structure of Pt occurred along with its structure and composition changes.

Among several catalytic mixtures, the PtCo@sOMC-900-1/3 catalyst showed an excellent performance. This catalyst exhibited an intermetallic compound structure, which resulted in exceptional kinetic activity and durability. The distribution of PtCo nanoparticles within the mesoporous structure of sOMC was highly dispersed, with only a small number of particles observed near the openings. The PtCo nanoparticles were effectively controlled to remain within a desirable particle size range of 3–5 nm, highlighting the impressive ability of the synthesized ordered MPC to confine the nanoparticles. Liao et al. [47] also demonstrated that the confinement of mesoporous carbon can efficiently control the particle size of Pt_3_Co and impede their aggregation during the thermal annealing process and electrochemical reaction support. The confinement environment was crucial in preventing the detachment of Pt_3_Co nanoparticles from the carbon support. This was an important factor that led to considerable improvements in the activity and durability of the Pt_3_Co/DMC-F catalyst. The summarized story highlights the critical role that mesoporous carbon serving as a nano-reactor plays in the development of strong, highly active PtCo alloy catalysts for ORR applications. 

Mesoporous carbon’s critical role in maintaining the robust performance of PtCo alloy catalysts, especially within high-current-density domains, even after rigorous durability testing, paves the way for its plausible adoption in heavy-duty vehicular applications. In-depth research was conducted by Elliot Padgett and colleagues investigating the impact of nanoscale shape on the functional efficacy of PtCo catalysts when housed on both readily available and conventional porous carbons like Ketjen Black [48]. The authors’ perceptive investigation revealed the existence of empty spaces within the porous carbon material, along with larger and more abundant mesopores, compared to traditional versions of the material. The local oxygen transport resistance was much lower than that of conventional porous carbon due to the augmented microporosity observed in the accessible porous carbon domain. The geometrical advantage greatly enhanced the efficiency of the catalyst in areas with high current density. The results of durability testing revealed a higher rate of electrochemically active surface area (ECSA) degradation for PtCo supported on porous carbons that are easily accessible. However, it is important to note that there was a significant retention of excellent performance at high current densities observed at the end of the testing period. This can be attributed to the improved local oxygen transport properties of the catalyst [26]. This significant discovery was additionally supported by a thorough review of data on oxygen-limiting currents. PtCo intermetallics, when supported on mesoporous carbon, exhibit notable characteristics that make them a highly viable option for utilization in heavy-duty vehicle applications. This statement highlights a fresh interpretation of the interplay between nanoscale morphology and mesoporous carbon support, which has propelled PtCo catalysts to the forefront of durable and highly efficient fuel cell applications in heavy-duty automotive vehicles.

## 4. Conclusions and Perspective

The widespread implementation of fuel cells in automotive applications is mostly impeded by the significant financial investment required and the substantial reliance on Pt resources. Utilizing mesoporous carbon as a catalytic support offers a revolutionary approach due to its exceptional efficiency. This accomplishment encompasses two significant achievements: firstly, a noteworthy decrease in the required amount of platinum, resulting in cost reduction, and secondly, a remarkable improvement in the performance and longevity of the catalyst. In a more specific context, MPC material effectively separates Pt from sulfonic acid groups, thereby reducing the occurrence of poisoning and enhancing the efficiency of local mass transportation, even under conditions of high current densities and low Pt loadings. Moreover, the distinctive characteristics of PtCo intermetallics allow them to retain structured atomic configurations, and thus they exhibit resistance to sintering even when subjected to elevated annealing temperatures.

MPC can enhance the transport of O_2_ while concurrently preserving the high-current-density performance of PtCo catalysts, even under demanding durability tests, despite notable reductions in ECSA and mass activity. Although certain strategies at the system level, such as inducing oxygen depletion at the cathode during shutdown, can help reduce the corrosion of advanced materials, the sustained feasibility of heavy-duty vehicles necessitates advancements in catalyst-support combinations that exhibit minimal particle mobility and susceptibility to poisoning while also displaying high resistance to corrosion.

In order to effectively tackle the difficulties of performance and stability, it is imperative for future research endeavors to concentrate on the following:

1. Mesoporous materials should be developed that exhibit precise control over pore diameters and tortuosity to enhance both mass transport and catalytic activity. Also, additional methods should be explored for alternative durable materials that can effectively collaborate with mesoporous carbon, with the ultimate goal of further reducing the utilization of Pt.
2. One area of focus is the development of adaptive mechanisms inside the mesoporous carbon matrix, which can effectively adjust to variations in operating conditions to improve the stability. The corrosion resistance and durability of these novel catalyst–support combinations are being validated through long-term studies conducted under harsh fuel cell conditions.
3. The atomically ordered PtCo intermetallics present on mesoporous carbon have significant potential not only to reduce the amount of Pt but also to regulate the surface electronic structure, thereby improving the activity and stability of oxygen reduction reactions. It is expected to attract growing interest in the research community as a cutting-edge advancement in developing PEMFC technology.
4. By designing and optimizing structural parameters such as the surface morphology, crystal plane, and size of Pt alloys, their activity and utilization can be improved, thereby reducing the demand for platinum. For example, preparing catalysts in the shape of nanoparticles or nanowires can increase their surface area, and by controlling the polycrystalline structure of the catalyst, the exposure of active sites can be improved. Pt loading can be reduced for the scaled-up fabrication of Pt alloys without compromising the performance of PEMFCs.
